# The French Gaucher’s disease registry: clinical characteristics, complications and treatment of 562 patients

**DOI:** 10.1186/1750-1172-7-77

**Published:** 2012-10-09

**Authors:** Jérôme Stirnemann, Marie Vigan, Dalil Hamroun, Djazia Heraoui, Linda Rossi-Semerano, Marc G Berger, Christian Rose, Fabrice Camou, Christine de Roux-Serratrice, Bernard Grosbois, Pierre Kaminsky, Alain Robert, Catherine Caillaud, Roselyne Froissart, Thierry Levade, Agathe Masseau, Cyril Mignot, Frédéric Sedel, Dries Dobbelaere, Marie T Vanier, Vassili Valayanopoulos, Olivier Fain, Bruno Fantin, Thierry Billette de Villemeur, France Mentré, Nadia Belmatoug

**Affiliations:** 1 INSERM, UMR 738, Laboratoire de Biostatistiques Hôpital Bichat, Assistance PubliqueHôpitaux de Paris (AP–HP), Paris, France; 2 Univ Paris-Diderot, Sorbonne Paris Cité, Paris, France, INSERM, UMR, Paris, 738, France; 3 Referral Center for Lysosomal Diseases (RCLD), Paris, France; 4 Hôpitaux Universitaires Paris Seine–Saint-Denis, AP–HP Service de Médecine Interne, Hôpital Jean-Verdier, Université, Paris XIII, Bondy, France; 5 Laboratoire de Génétique Moléculaire CHU Montpellier, Hôpital Arnaud-de-Villeneuve, Montpellier, France; 6 Service de Médecine Interne, Hôpital Beaujon, AP–HP, Clichy, France; 7 Service de Pédiatrie et Pédiatrie Rhumatologique, Hôpital de Bicêtre, AP–HP, National Reference Center for Auto-Inflammatory Diseases, Université de Paris Sud, Le Kremlin–Bicêtre, France; 8 Service d'Hématologie Biologique–Immunologie, CHU Estaing, Clermont-Ferrand, France; 9 Service d’Hématologie, Hôpital Saint-Vincent-de-Paul, Lille, France; 10 Service de Réanimation Médicale, CHU Saint-André, Bordeaux, France; 11 Service de Médecine Interne, Hôpital Saint-Joseph, Marseille, France; 12 Service de Médecine Interne, Etablissements Nord Sud, Site Hôpital Sud, Rennes, France; 13 Service de Médecine Interne, CHU de Nancy, Hôpitaux de Brabois, Vandoeuvre, France; 14 Service de Pédiatrie, Hôpital des Enfants, Toulouse, France; 15 Laboratoire de Génétique, Hôpital Cochin, Paris, France; 16 Centre de Biologie Est, Hospices Civils de Lyon, Bron, France; 17 Laboratoire de Biochimie Métabolique, Institut Fédératif de Biologie, CHU Purpan, Toulouse, France; 18 Service de Médecine Interne, CHU Hôtel-Dieu, Nantes, France; 19 Unité Fonctionnelle de Génétique Clinique, Groupe Hospitalier Pitié–Salpêtrière, AP–HP, Paris, France; 20 Service de Neuropédiatrie et Pathologie du Développement, Hôpital Armand-Trousseau, AP–HP, Université Pierre-et-Marie-Curie UPMC, Paris, France; 21 Département de Neurologie, Hôpital Pitié–Salpêtrière, AP–HP, Paris, France; 22 Centre de Référence des Maladies Héréditaires du Métabolisme de l’Enfant et de l’Adulte, Hôpital Jeanne-de-Flandre, Lille, France; 23 INSERM U 820, Faculté de Médecine Lyon-Est Claude-Bernard, Lyon, France; 24 Centre de Référence Maladies Métaboliques de l'Enfant et de Adulte (MaMEA), Hôpital Necker-Enfants Malades et Université Paris, Descartes, Paris, France; 25 Hôpitaux Universitaires de Genève, Service de Médecine Interne Générale, Rue Gabrielle-Perret-Gentil 4, CH-1211, Genève 14, Suisse

**Keywords:** French Gaucher’s Disease Registry, Bone events, Enzyme-replacement therapy

## Abstract

**Background:**

Clinical features, complications and treatments of Gaucher’s disease (GD), a rare autosomal–recessive disorder due to a confirmed lysosomal enzyme (glucocerebrosidase) deficiency, are described.

**Methods:**

All patients with known GD, living in France, with ≥1 consultations (1980–2010), were included in the French GD registry, yielding the following 4 groups: the entire cohort, with clinical description; and its subgroups: patients with ≥1 follow-up visits, to investigate complications; recently followed (2009–2010) patients; and patients treated during 2009–2010, to examine complications before and during treatment. Data are expressed as medians (range) for continuous variables and numbers (%) for categorical variables.

**Results:**

Among the 562 registry patients, 265 (49.6%) were females; 454 (85.0%) had type 1, 22 (4.1%) type 2, 37 (6.9%) perinatal–lethal type and 21 (3.9%) type 3. Median ages at first GD symptoms and diagnosis, respectively, were 15 (0–77) and 22 (0–84) years for all types. The first symptom diagnosing GD was splenomegaly and/or thrombocytopenia (37.6% and 26.3%, respectively). Bone-marrow aspiration and/or biopsy yielded the diagnosis for 54.7% of the patients, with enzyme deficiency confirming GD for all patients. Birth incidence rate was estimated at 1/50,000 and prevalence at 1/136,000. For the 378 followed patients, median follow-up was 16.2 (0.1–67.6) years. Major clinical complications were bone events (BE; avascular necrosis, bone infarct or pathological fracture) for 109 patients, splenectomy for 104, and Parkinson’s disease for 14; 38 patients died (neurological complications for 15 type-2 and 3 type-3 patients, GD complications for 11 type-1 and another disease for 9 type-1 patients). Forty-six had monoclonal gammopathy. Among 283 recently followed patients, 36 were untreated and 247 had been treated during 2009–2010; 216 patients received treatment in December 2010 (126 with imiglucerase, 45 velaglucerase, 24 taliglucerase, 21 miglustat). BE occurred before (130 in 67 patients) and under treatment (60 in 41 patients) with respective estimated frequencies (95% CI) of first BE at 10 years of 20.3% (14.1%–26.5%) and 19.8% (13.5%–26.1%).

**Conclusion:**

This registry enabled the epidemiological description of GD in France and showed that BE occur even during treatment.

## Background

Gaucher’s disease (GD), a rare autosomal–recessive disorder with an approximate prevalence of 1/75,000 live births worldwide, is due to the deficiency of a lysosomal enzyme (glucocerebrosidase, glucosylceramidase or glucosidase-β acid (EC 3.2.1.45)) [1] or, more rarely, its activator (saposin C) [2,3]. GD diagnosis is based on deficient glucocerebrosidase activity in peripheral leukocytes or cultured skin fibroblasts. Genotyping can sometimes provide prognostic information [4]. This lysosomal storage disease is characterized by liver and spleen enlargement, and bone manifestations (Erlenmeyer-flask deformity, osteoporosis, lytic lesions, pathological and vertebral compression fractures, bone infarcts and avascular necroses leading to degenerative arthropathy) [1,5]. Based on the neurological signs, 3 clinical phenotypes are recognized: type 1, the classic form usually defined by the absence of central nervous system impairment, although an association between type-1 GD and Parkinsonism has been described [6]; types 2 and 3, both rare and severe, have neurological involvement [7]; and the perinatal–lethal GD type, with perinatal onset and death before 3 months of age [8]. GD signs usually appear after a symptom-free period, except in rare cases of fetal onset [9]. Thrombocytopenia and anemia are common, and several biomarkers (chitotriosidase, ferritin, angiotensin-converting enzyme [ACE] and tartrate-resistant acid phosphatase [TRAP]) are elevated during GD evolution [10-17].

Enzyme-replacement therapy (ERT; alglucerase [Ceredase©, Genzyme Corporation, available since 1991] [18], followed by imiglucerase [Cerezyme©, Genzyme Corporation, available since 1996], velaglucerase [Vpriv©, Shire, available since 2010] [19], and taliglucerase [Uplyso©, Pfizer, only authorized for temporary use] [20]), is the reference treatment. Substrate-reduction therapy (SRT), namely miglustat (Zavesca©, Actelion, available since 2002) [21], is indicated for moderate GD when ERT is unsuitable. In June 2009, an acute imiglucerase shortage occurred because of viral contamination (Vesivirus 2117) of cell cultures and other production problems [22]. Since then, that ERT has been in short supply, which was further aggravated in August 2009.

Genzyme Corporation developed an international registry [23] and several countries, e.g., Spain [24,25], Brazil [26] or Japan [27], identified GD cohorts and established exhaustive national registries. While the international registry conducted many important ancillary studies [28-34], its non-exhaustive cohort did not address public health issues in terms of incidence, prevalence and monitoring of care of GD patients. Since 2004, France has created referral centers dedicated to the clinical management of rare diseases, and assigned them several objectives, e.g., improving overall patient clinical care and professional practices, and collecting epidemiological data. In this context, a Referral Center for Lysosomal Diseases (RCLD) was established and a national GD-patient registry was created, in 2009, as a means to examine and meet some of those goals.

The main aims of this study were to describe the epidemiological profile of GD patients in France: GD demographic, clinical, biological and genetic features; complications in patients with follow-up (2009–2010); and treatments for those with recent (2009–2010) follow-up based on data collected since 1980 and available in the French Gaucher Disease Registry (FGDR).

## Patients and methods

### Registry design

The FGDR developed and maintains a designated RCLD since 2009. Its Evaluation of Gaucher-Disease–Treatment Committee (EGDTC) is a national scientific committee to monitor and optimize GD management in France. The French Data-Protection Commission’s (CNIL) approval of the FGDR required oral or written informed consent from patients or their parents. Data from patients who did not consent were not entered. The FGDR was finally certified in 2009 by the French Institute for Public Health Surveillance (InVS) and the French National Institute of Health and Medical Research (INSERM). All GD patients living in France and having ≥1 consultations (i.e., hospitalization or outpatient consultation with a GD specialist) since 1980 were included. For all patients, GD was diagnosed by demonstration of deficient glucocerebrosidase activity in leukocytes [35] or cultured skin fibroblasts. Exhaustive identification of cases was achieved through 3 sources. Only 3 diagnostic laboratories (all included in EGDTC) are accredited in France and, therefore, identify all patients with an enzymatic assay for GD. Based on our Reference Center’s expertise, the French national health insurance (Rare Disease Committee with EGDTC members) validates each GD diagnosis and authorizes coverage for its treatment. Once experts have validated the case, they can ask the treating physician to include the patient in our registry. The indications for treatment are well established to maximize efficacy and avoid unnecessary health insurance expenditures. Each treating physician contacted allowed access to the medical data entered in the FGDR, which is certified by InVS and INSERM.

Each patient’s data were also collected by RCLD physicians or clinical research assistants. The FGDR director controlled data quality. Dr D. Hamroun developed the original Internet software for the FGDR, using 4^th^ Dimension language from 4D ( http://www.4D.com). Data were collected retrospectively between 2009 and 2010, and as of 2011, all data have been recorded prospectively.

A standardized case-report form was used to collect the following information: initial data (age at diagnosis, sex, history related or unrelated to GD, symptoms leading to diagnosis and first symptoms, first diagnostic exam, phenotype, genotype and affected family members); clinical information during the first consultation, at diagnosis and throughout follow-up; body mass index expressed according to the World Health Organization classification; organomegaly (liver and/or spleen, ultrasound measurement of the largest diameter); biological findings initially and throughout follow-up (hemoglobin level, platelet count, leukocyte count, chitotriosidase, ferritin, ACE, TRAP, gammaglobulin (with respective normal values of >12 g/dL, >150×10^3^/mm^3^, >4 × 10^3^/mm^3^, <100 nmol/mL/h, <250 ng/L, <45 IU/L, <7 IU/L and <13 g/L). Plasma chitotriosidase activity was determined using the fluorescent substrate 4-methylumbelliferylβ-D-*NN*′,*N*′′-triacetylchitotriose [10]; ACE, TRAP, ferritin and other markers were measured in the appropriate local laboratories. Bone findings (X-rays, magnetic resonance imaging and, for some patients, scintigraphy and dual-energy X-ray absorptiometry) were recorded during follow-up, with identification of intercurrent events, particularly bone complications. Bone events (BE) were defined clinically, using the bone indications for treatment recommended by the French National Health Authority [36]: avascular necrosis of an epiphysis, bone infarct, pathological and/or vertebral compression fracture(s). Each BE caused a clinical manifestation and was confirmed radiologically. Bone pain alone was not considered a BE without radiological confirmation. Acute bone pain defined a bone crisis. Bone crisis was included in BE only when a bone infarct was identified. Any event, GD-related or not, occurring during follow-up and monitoring of GD-specific therapy was also recorded.

### Study design

This investigation was undertaken to describe and analyze clinical, biological, radiological and therapeutic data recorded in the FGDR for all patients from diagnosis until 31 December 2010, the closing date. The local Institutional Review Board of Northern Paris Hospitals, Paris–Diderot University, AP–HP (Ethics Committee) reviewed and approved the research project.

To simplify the description, we defined 4 groups: the entire cohort and its subgroups. Data from the entire cohort of patients entered in the FGDR described, when available, diagnosis characteristics for these patients, the GD-incidence rate (defined as total number of cases diagnosed between 1980 and 2010 divided by the total French population during the same period), birth incidence rate (defined as the total number of cases diagnosed between 1980 and 2010, divided by the total number of live births during the same period) and prevalence were estimated for the French population. For patients with ≥1 follow-up visits in addition to the initial assessment form, we investigated their GD complications (splenectomy, Parkinson’s disease (PD), monoclonal gammopathy (MG), BE, first treatment and deaths). Recently followed patients had consulted in 2009–2010: a map showing the locations of hospitals monitoring them was drawn. For patients seen and treated in 2009–2010, BE were analyzed, before and under (ERT and/or SRT (ERT/SRT)). Clinical, biological and radiological monitoring of these recently treated patients was also investigated. Specific GD ERT (imiglucerase, velaglucerase, taliglucerase, miglustat) was studied, particularly during the period of imiglucerase shortage (June 2009–December 2010). Patients were distinguished according to their age on 1 June 2009 (≤15 or >15 years) for the description of the shortage that began at that time. That age was chosen because it defines the limit between adult medicine and pediatrics.

### Statistical analyses

All statistical analyses were computed with SAS software (version 9.2; SAS Institute Inc; Cary, NC). Data are expressed as medians ((range) or interquartile range [IQR; Q1;Q3]) for continuous variables and numbers (%) for categorical variables. Because this was a retrospective study, some data were missing, particularly at the onset of follow-up (during the diagnosis phase) or at treatment onset. Given the demonstrated relatively stable clinical and laboratory parameters of untreated patients after GD diagnosis [37], the biological data during the next 2 years changed only minimally and were considered similar to those at diagnosis. Likewise, data for the previous 2 years under ERT/SRT were stable compared to those at treatment onset. Under treatment data were the last values before the end of therapy or at the closing date. When ERT/SRT was interrupted for <6 months, patients were always considered to be on treatment.

Non-parametric tests were used to compare categorical variables (Fisher’s exact test) across patient subgroups. A two-sided p<0.05 was considered significant. For recently treated patients, time to first BE was estimated with the Kaplan–Meier method for 2 periods: diagnosis to treatment onset (before ERT) and first ERT to closing date (under ERT), with only the first BE occurring during each period being considered. Data were censored when no BE occurred prior to ERT start for the first analysis, and until the closing date or treatment discontinuation for the second. We aimed to study a risk effect of BE. First, the impact of splenectomy, time to treatment onset (< or ≥2 years after diagnosis, based on Mistry et al.’s demonstration of lower BE risk after the latter [38]) or age at diagnosis (≤ or >15 years) on BE occurrence was tested using the log-rank test; second a Cox model was used to derive a predictive model. When several univariate model covariates were significant, a multivariate Cox model with backward selection was used to retain only significant ones. For BE under ERT, the impact of BE occurrence before treatment was also tested.

## Results

### Entire registry cohort

The FGDR contained 562 patients with confirmed GD, living in France and having ≥1 consultations between 1980 and 2011. Patient characteristics at diagnosis are reported in Table 1. The male/female ratio was 1.0151 (49.6% female), while median age at first symptoms was 15 (0–77) years. During the 31 years (1980–2010), 474 patients were diagnosed in France, with an annual median of 15 (9–32) patients. The incidence rate was estimated at 0.26 patients/10^6^ person-years and birth incidence at 1/50,000. The prevalence was 1/136,000. The most frequent first symptom leading to diagnosis (available for 232 patients) was splenomegaly and/or thrombocytopenia, although others were found, including anemia in 14, 14 splenectomies, 10 severe hemorrhages, 6 neurological symptoms, 3 avascular necroses, 1 vertebral collapse and 1 bone infarct. Although the most common diagnostic test yielding the diagnosis was bone-marrow aspiration, all patients’ diagnoses were confirmed by enzymatic assay. Genotypes were determined for 261 patients, with 203 having homo- (39 patients) or heterozygous p.*N370S* mutations (164 patients, including 41 with p.*L444P*/p.*N370S* mutations). Genotype distributions differed significantly (p<0.0001): *L444P*/*L444P* or *L444P*/other were found in 14 (6.4%) phenotype-1, 13 (100%) phenotype-2 and 12 (100%) phenotype-3 patients, while the genotypes *N370S*/*L444P*, *N370S*/other or *N370S*/*N370S* were identified in 203 (93.6%) phenotype-1, no phenotype-2 and no phenotype-3 patients. Other mutations found (associated or not with p.*N370S* or p.*L444P*) were: p.*R48W*, p.*C16W*, p.*G193R*, p.*K303I*, p.*W312S*, p.*G377S*, p.*W393R*, p.*V394L*, p.*D409H*, p.*M416I*, p.*A446P*, p.*R463C*, p.*1416*, *1417delAG* and p.*RecNcil*. Patients were predominantly (84.7%) type 1. Among 161 patients with an affected family member, all but 3 (1 mother, 1 uncle, 1 cousin) were siblings. Ninety-seven patients died: 37 perinatal deaths (28 fetuses and 9 newborns <3 months old) and 60 later (33 type 1, 22 type 2 and 5 type 3).

**Table 1:** Baseline characteristics of the FGDR cohort, and subgroups with any follow-up, recent follow-up or treatment

**Baseline characteristic**	**Entire cohort**		**Followed patients**		**Recently seen patients**		**Recently treated patients**	
	**(n=562)**		**(n=378)**		**(n=283)**		**(n=247)**	
	No.*	**Value**	No.*	**Value**	No.*	**Value**	**No.**	**Value**
Sex, n (%)	562		378		283		247	
Female		265 (49.6)		182 (48.1)		144 (50.9)		121 (49)
Male		269 (50.4)		196 (51.9)		139 (49.1)		126 (51)
Fetuses		28						
Age, years, median (range) [IQR]								
First symptom(s)	238	15 (0–77) [5;30]	227	15 (0–77) [5;30]	182	15 (0–62) [5;30]	162	15 (0–62) [5;29]
Diagnosis (without fetuses)	534	22 (0–83.8) [5.8;38.9]	378	21.9 (0–80.5) [6.8;36.2]	283	22.6 (0.2–67.5) [8.4;35.1]	247	22.1 (0.5–67.5) [8.7;37.7]
Patients diagnosed before 1991, n (%)	562	261 (46.4)	378	181 (47.9)	283	131 (46.3)	247	122 (49.4)
Patients ≤15 years old at diagnosis, n (%)	562	245 (43.6)	378	147 (38.9)	283	102 (36.0)	247	125 (50.6)
1^st^ symptom-to-diagnosis interval, years, median (range)	238	1 (0–56)	227	1 (0–56)	182	1 (0–56)	162	1 (0–56)
First symptoms, n (%)†	232		216		169		143	
Splenomegaly		163 (70.3)		155 (71.8)		120 (71)		109 (38.9)
Hepatomegaly		51 (22)		46 (21.3)		35 (20.7)		33 (11.8)
Thrombocytopenia		114 (49.1)		107 (49.5)		88 (52.1)		76 (27.1)
Bone crisis		8 (3.4)		8 (3.7)		6 (3.6)		6 (2.1)
Chronic bone pain		16 (6.9)		15 (6.9)		13 (7.7)		13 (4.6)
Other		82 (35.3)		73 (33.8)		53 (31.4)		43 (15.5)
Test diagnosing GD, n (%)‡	245		233		189		162	
Enzyme assay		61 (24.9)		53 (22.7)		48 (25.4)		36 (22.2)
*GBA*-gene sequencing		1 (0.4)		1 (0.4)		1 (0.5)		1 (0.6)
Bone-marrow aspiration		118 (48.2)		115 (49.4)		93 (49.2)		83 (51.3)
Bone-marrow biopsy		16 (6.5)		16 (6.9)		13 (6.9)		13 (8)
Bone biopsy		5 (2.0)		5 (2.1)		5 (2.6)		5 (3.1)
Hepatic biopsy		9 (3.7)		8 (3.4)		6 (3.2)		4 (2.5)
Spleen histology		33 (13.5)		33 (14.2)		21 (11.1)		18 (11.1)
Other		2 (0.8)		2 (0.9)		2 (1.1)		2 (1.2)
Type, n (%)	536		378		283		247	
1		454 (84.7)		348 (92.0)		274 (96.8)		239 (96.8)
2		61 (11.4)		15 (4)		1 (0.4)		
3		21 (3.9)		15 (4)		8 (2.8)		8 (3.2)
Genotype, n (%)	261		229		172		155	
p.*N370S*/p.*N370S*		39 (15.0)		34 (14.8)		28 (16.3)		24 (15.5)
p.*N370S*/p.*L444P*		41 (15.7)		37 (16.2)		31 (18.0)		27 (17.4)
p.*L444P*/p.*L444P*		17 (6.5)		11 (4.8)		4 (2.3)		4 (2.6)
p.*N370S*/other		123 (47.1)		114 (49.8)		86 (50)		77 (49.7)
p.*L444P*/other		24 (9.2)		17 (7.4)		9 (5.2 )		9 (5.8)
Other/other		17 (6.5)		16 (7)		14 (8.2)		14 (9)
Affected family, n	562	161	378	130	283	108	247	94

Note that recent refers to 2009–2010, i.e., the last 2 years. *GBA* glucosidase-β acid.

*No. represents the number of patients with available information.

†Several symptoms for each patient.

‡All patients had their definitive diagnoses confirmed by enzymatic assay.

### Followed patients

A total of 378 patients, predominantly type 1, had ≥1 follow-up visits after their initial evaluations. The median follow-up duration was 16.2 (0.1–67.6) years. Their characteristics at diagnosis are reported in Table 1. During follow-up, 225 complained of chronic bone pain or clinical bone crisis, 231 had splenomegaly and 163 had hepatomegaly at least once.

#### Splenectomy

Surgery was performed on 104 patients: 17 before diagnosis, 46 at diagnosis and 41 >1 year postdiagnosis. Among the 41 patients splenectomized after diagnosis, 27 had the surgery before 1991 and only 14 thereafter. Among the 14 splenectomies after 1991, indications were: 2 for refractory idiopathic thrombocytopenic purpura, 2 for splenic rupture, 2 for major hypersplenism, 2 for severe splenic infarct and 2 for splenic hematoma but 4 had wrong or unknown indications. Splenectomy was more frequent in type 1 (29.3%) than type 3 patients (2, 13.3%) (p=0.009). Median age at splenectomy was 24.6 (3–76) years. Only 3 patients were taking ERT/SRT when splenectomy was performed for splenic complications.

#### BE

One hundred and nine patients experienced BE during follow-up, with a median of 1 (1–8) BE per patient and a total of 223 BE: 89 avascular necroses, 40 bone infarcts, 67 pathological fractures and 27 vertebral collapses. Sites were known for 188 BE: 56 avascular necroses affected the femur, 8 the humerus, 5 the tibia and 7 other bones (astragal, iliac crest, calcaneus), with 13 unknown sites; 15 bone infarcts involved the femur, 5 the tibia, 5 the iliac crest and 2 other bones, with 13 sites unknown; 11 pathological fractures concerned the wrists, 9 the ribs, 8 the femur, 8 the humerus and 22 other bones, with 9 localizations unknown. Type-1 patients had 107 (30.7%) BE, type-3 patients had 2 (13.3%), while type-2 patients had no BE (p=0.009). Median age at first BE was 34.1 (0–75.9) years. The first BE occurred in 88 patients without treatment and in 22 under ERT/SRT.

#### MG

Forty-six patients, all type 1, developed MG during follow-up. Median age at MG diagnosis was 48.2 (24.3–81) years. The median GD-diagnosis-to-MG-diagnosis interval was 1.3 (0–47.5) years. MG was not significantly more frequent in any given genotype (p=0.37): 1 (4%) p.*N370S*/p.*N370S*, 15 (60%) p.*N370S*/other, 6 (24%) p.*N370S*/p.*L444P*, 1 (4%) p.*L444P*/other and 2 (8%) in other/other; 21 patients with MG had no genotype determination. Comparing the p.*L444P* allele to the others, no significant difference was found (p=1). Thirteen patients were on ERT/SRT when MG was diagnosed (but no pretreatment immunofixation was available), 23 were treated thereafter and 10 had never been treated at closing date, despite MG.

#### PD

Neurologist-confirmed PD was diagnosed in 14 patients, all had type-1 GD; 6 had dementia. Median age at PD diagnosis was 60.4 (38.2–77.1) years. The median age at GD diagnosis of patients who developed PD was 46.2 (4.4–70.7) years. PD was significantly more frequent in patients with the p.*L444P*/p.*N370S* mutation versus others (p<0.0001): PD was diagnosed in 1 (2.9%) p.*N370S*/p.*N370S* patient, 2 (1.7%) p.*N370S*/other and 5 (13.5%) p.*N370S*/p.*L444P*; 6 PD patients had no genotype determination. Five patients were on ERT/SRT at PD diagnosis, 7 were treated thereafter and 2 had never been treated at closing date, despite PD. Treatment (imiglucerase or miglustat) did not have any apparent effect on PD signs. Two patients received miglustat and both stopped it after 1 year because of PD progression or dementia.

#### Treatments

Among the 378 patients with follow-up, 298 (78.8%) received treatment. The first ERT was alglucerase for 62 (20.8%) patients, imiglucerase for 224 (75.2%), miglustat for 7 (2.3%), velaglucerase for 4 (13.4%) and taliglucerase in 1 (0.3%). The median ERT dose (known for 228 patients) was 120 (30–284) IU/kg/month, with 204 patients receiving ≥120 IU/kg/month. Sixty-two patients were given miglustat during follow-up, with a median treatment duration of 0.7 (0.1–6.7) years. Ten patients had an indwelling catheter and 85 received ≥1 ERT administrations at home. The median diagnosis-to-first-treatment interval was 9.1 (0–61.4) years for all 298 patients, but only 1.4 (0–16) years for the 145 patients diagnosed after 1991.

#### Deaths

Thirty-eight patients died during follow-up: type 2 was fatal for all 15 patients compared to 20 (5.7%) type 1 or 3 (20%) type 3 (p<0.001). Age at death was known for 28 patients: median of 8.4 (0.3–83.4) years for all deceased, but 64.5 (38.4–83.4) years for 13 type-1 patients, 1 (0.3–2.2) year for the 13 type-2 patients and 8.4 (7.2–9.6) years for the 2 type-3 patients. Type-2 and −3 patients died of neurological impairment, whereas 11 type-1 patients’ deaths were attributed to: 2 lymphomas, 3 PD, 1 myeloma, 1 osteosarcoma, 2 with anemia or thrombocytopenia complications (1 each with myocardial infarct with anemia or pancytopenia) and 2 had pulmonary hypertension. Mortality was significantly more frequent (p<0.0001) for patients whose genotypes carried the p.*L444P* mutation; most of them had neurological impairment, as reported above: 1 (2.9%) with p.*N370S*/p.*N370S*, 3 (2.6%) p.*N370S*/other, 4 (10.8%) p.*N370S*/p.*L444P*, 5 (31.2%) p.*L444P*/other and 6 (54.5%) p.*L444P*/p.*L444P*; 17 deceased patients had no genotype determination. Fifteen patients were on ERT/SRT when they died, while all others had never been treated (including all type-2 patients).

### Recently followed patients

Among followed patients, 95 had no known data during 2009–2010. Median time since the last hospital consultation for these patients was 10.7 (2.4–31.6) years. Thus, 283 patients had consulted during 2009–2010: 36 had never been treated and 247 patients had received ERT/SRT during that period. Their characteristics at diagnosis are reported in Table 1.

#### Hospital locations of followed patients

**Figure 1:**
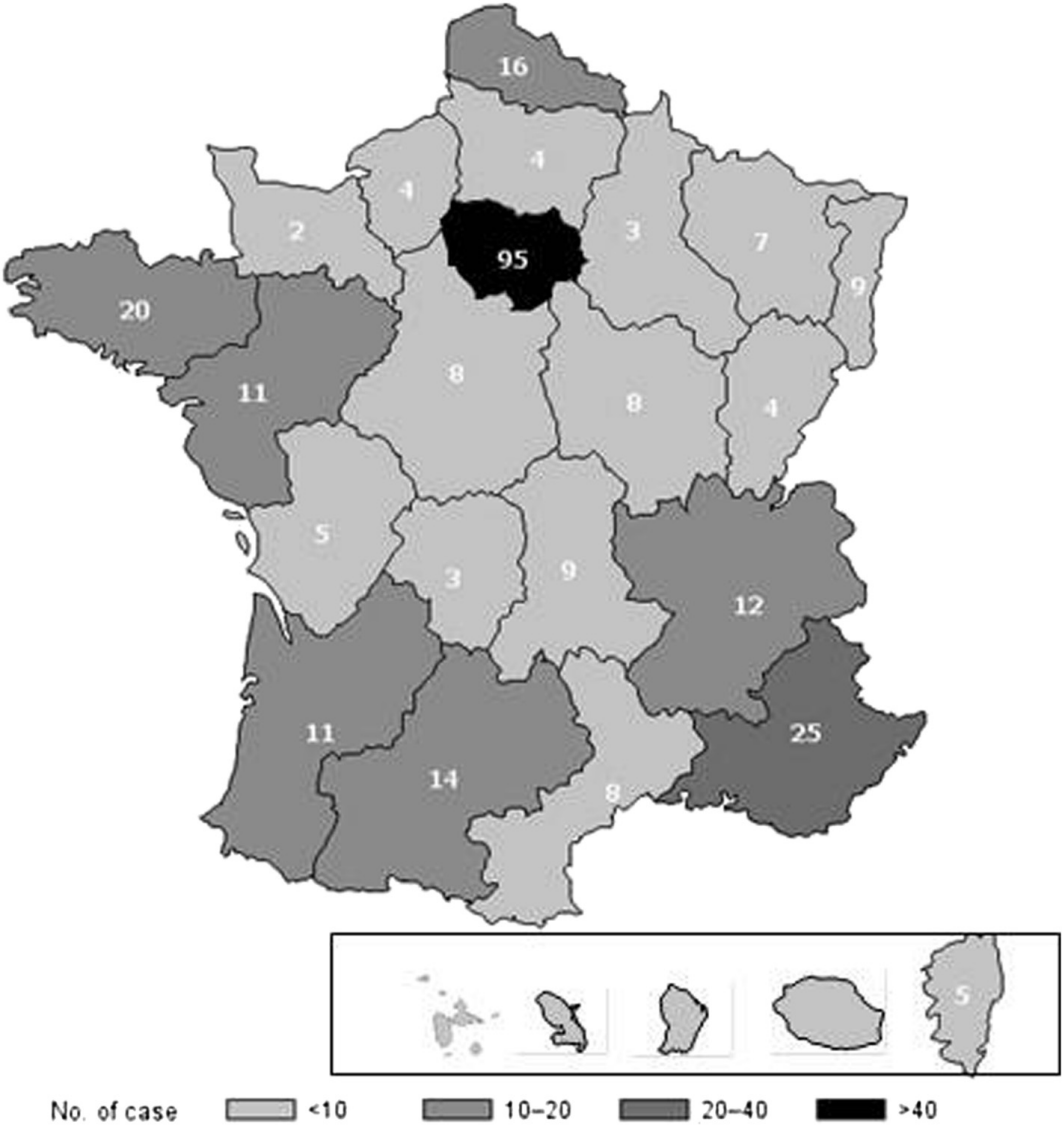
Map showing the locations of the hospitals monitoring the 283 patients with recent follow-up (2009–2010).

Figure 1 shows where the hospitals caring for the 283 patients with recent follow-up are located. Ninety-five patients were followed in Paris, including 73 (76.8%) in the RCLD. Follow-up was done in an internal medicine department for 152 (54%) patients, hematology for 65 (23%), pediatrics for 30 (11%) and other departments (7 gastroenterology, 6 neurology, 10 rheumatology, 13 others) for 36.

#### Patients without treatment

Thirty-six patients had never been treated: median diagnosis-to-closing-date interval was 7.8 (0.4–39.9) years, for 35 type-1 patients and 1 with type 2; 14 patients had familial GD (all siblings) and 3 patients, who developed BE (2 avascular necroses, 1 pathological fracture and 1 vertebral collapse) during follow-up, refused treatment.

### Recently seen and treated patients

During 2009–2010, 247 patients (239 type 1 and 8 type 3) received treatment, with median follow-up at 19.3 (0.2–66.2) years. Among these recently treated patients, all 5 type-1 patients died at a median age of 65.8 (56.8–83.4) years. Table 2 provides clinical, biological and bone data at diagnosis, treatment onset and closing date. Clinical findings, biological values and bone findings tended to improve under ERT/SRT.

**Table 2:** GD clinical, biological and imaging characteristics at specific times for 247 recently treated (2009–2010) patients

**Characteristic**	**No.**	**At diagnosis**	**No.**	**At ERT/SRT onset**	**No.**	**At closing date**
Years since diagnosis, median (range) [IQR]	247	–	247	9.2 (0–47) [1.5;17.7]	247	17.6 (0.1–66.2) [9.3;26.2]
Age, years, median (range) [IQR]	247	22.2 (0.5–67.5) [8.7;34.7]	247	36 (1–79) [20.9;48.2]	247	43.4 (3.2–82.4) [29.9;56.7]
Clinical involvement, n (%)*						
Pigmentation	184	10 (5.4)	186	14 (7.5)	167	1 (0.6)
Asthenia	184	84 (45.7)	186	106 (57)	167	44 (26.3)
Abdominal pain	184	29 (15.8)	186	43 (23.1)	167	8 (4.8)
Chronic bone pain	184	70 (38.0)	186	79 (42.5)	167	41 (24.6)
Bone crisis	184	25 (13.6)	186	46 (24.7)	167	12 (7.2)
Bleeding	184	57 (31)	186	58 (31.2)	167	12 (7.2)
Neurological sign	184	7 (3.8)	186	14 (7.5)	167	9 (5.4)
Other	184	11 (6)	186	10 (5.4)	167	42 (25.1)
Body mass index, kg/m^2^, median (range)	49	16.6 (13.6–28.1)	78	20.3 (13.6–28.1)	53	22.2 (14.6–34.4)
Underweight, n (%)		31 (62.3)		31 (39.7)		7 (13.2)
Normal, n (%)		14 (28.6)		41 (52.6)		35 (66.0)
Overweight/obese, n (%)		4 (8.1)		6 (7.7)		11 (20.8)
Liver and spleen*						
Splenectomy, n (%)	247	41 (16.6)	247	63 (25.5)	247	65 (26.3)
Splenomegaly†, n (%)	176	174 (98.9)	129	124 (96.1)	76	42 (55.3)
Splenic US, median (range) of largest diameter, cm	54	15.8 (10–32)	86	18.9 (10–41)	44	13.6 (8–24)
Hepatomegaly, n (%)	146	116 (79.5)	140	118 (84.3)	90	40 (44.4)
Liver US, (median (range) of largest diameter, cm	23	15 (8.4–22)	81	17.6 (8.4–37)	34	15 (9–22)
Biological parameter, median (range)†						
Hemoglobin (g/dL)	140	11.5 (5.3–18.9)	169	11.7 (5.4–17)	188	13.2 (8–16.4)
Leukocyte (×10^3^/mm^3^)	126	4.9 (0.6–15.4)	153	4.8 (0.5–24)	102	5.7 (2.1–14.1)
Platelet count (×10^3^/mm^3^)	161	81 (20–420)	185	80 (18–449)	187	160 (18–553)
Platelets (×10^3^/mm^3^) without splenectomy	127	80 (20–246)	137	72 (18–189)	144	139 (18–304)
Chitotriosidase (nmol/mL/h)	43	8,900 (239–47,500)	71	9,000 (360–85,500)	106	992 (19–53,400)
TRAP (IU/L)	5	7.1 (1.1–28)	29	10 (4–38)	24	4.5 (1–18.8)
ACE (IU/L)	17	183 (93–1000)	51	190 (3.4–450)	48	57.5 (12–380)
Ferritin (ng/L)	36	500 (40–5000)	74	621 (63–3,230)	72	337 (40–2,200)
Gammaglobulin (g/L)	14	15.8 (9–28.7)	44	15 (6.6–36)	36	12 (5.4–19.9)
Imaging of bone lesions*, n (%)						
Erlenmeyer flask	43	9 (20.9)	61	17 (27.9)	50	4 (8)
Osteopenia	43	6 (14)	61	15 (24.6)	50	4 (8)
Cortical erosion	43	3 (7)	61	3 (4.9)	50	0
Lytic lesion	43	4 (9.3)	61	5 (8.2)	50	5 (10)
Avascular necrosis sequelae	43	6 (14)	61	11 (18.0)	50	4 (8)
Infarct sequelae	43	6 (14)	61	8 (13.1)	50	2 (4)
Fracture sequelae	43	0	61	2 (3.3)	50	1 (2)
Infiltration on MRI	40	31 (77.5)	72	53 (73.6)	80	40 (50)
^99m^Tc-Hyperfixation	31	19 (61.3)	56	42 (75)	41	30 (73.2)
^99m^Tc-Hypofixation	31	5 (16.1)	56	5 (8.9)	41	0
Bone densitometry, median (range)						
T-score neck	10	−0.6 (−2.1 to 1.1)	27	−1.4 (−4.2 to 1.4)	28	−0.6 (−2.9 to 4.5)
T-score lumbar	8	−1.5 (−2.8 to −0.5)	22	−1.8 (−4.2 to 0.8)	29	−0.9 (−3.0 to 6.2)
Z-score neck	10	−0.8 (−2.1 to 1)	20	−0.8 (−2.1 to 1.9)	26	−0.5 (−2.6 to 4.4)
Z-score lumbar	7	−1.9 (−3 to 0.3)	15	−1.1 (−3.1 to 0.5)	24	−0.1 (−3.0 to 7.1)

*US* ultrasound, *MRI* magnetic resonance imaging.

*Data from 31 patients were used at diagnosis and at ERT/SRT onset.

†Splenomegaly in non-splenectomized patients.

**Figure 2:**
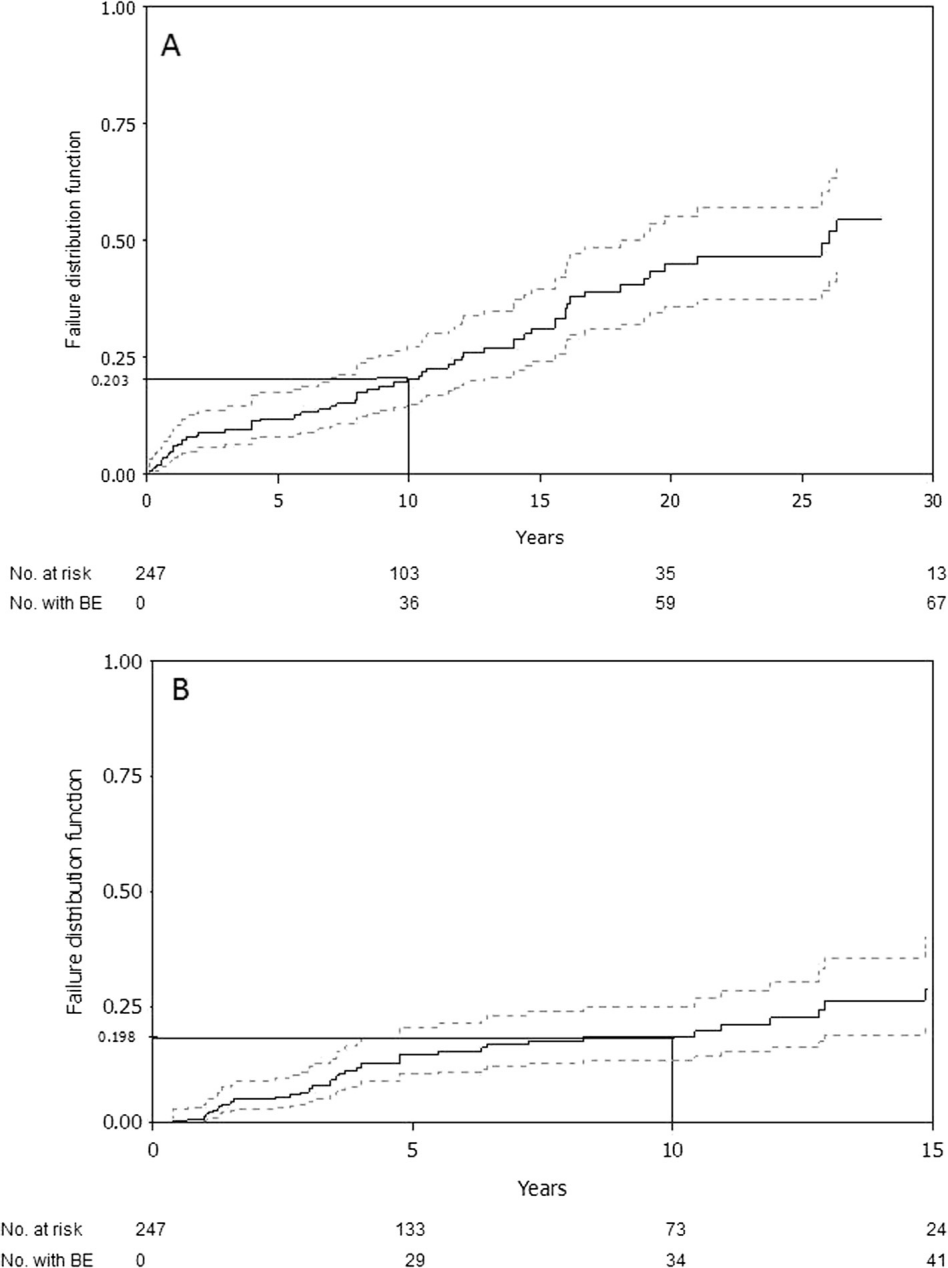
**Time to the first bone event (BE) in 247 GD patients receiving ERT/SRT.** The dashed lines represent the curve’s 95% CI; the estimated probability of BE occurrence after 10 years is reported on the y-axis. (**A**) Between diagnosis and first treatment during the first 30 years of follow-up. (**B**) Between first treatment and end of treatment or closing date during 15 years of follow-up. No. at risk represents the number of patients followed at the indicated time; No. with BE represents the number of patients with a BE.

During follow-up, 190 BE (73 avascular necroses, 36 bone infarcts, 58 pathological fractures, 23 vertebral compressions) occurred in 86 patients, with a median of 1.5 (1–8) BE/patient. Figure 2 shows Kaplan–Meier estimates of the time to the first BE for the 247 recently seen and treated patients, between diagnosis and ERT/SRT onset (9.2 years of follow-up), and between the latter and the closing date (7.8 years of follow-up). Treatment at the time of BE was imiglucerase for 56 patients, alglucerase for 5 and miglustat for 4. The median imiglucerase/alglucerase dose when BE occurred in 52 patients was 120 (43.5–240) IU/kg/month; 42 patients had doses ≥120 IU/kg/month. The probabilities (95% confidence interval [CI]) of BE occurring by 10 years before and during treatment were estimated at 20.3% (14.1%–26.1%) and 19.8% (13.5%–26.1%), respectively. Before treatment, 67 patients developed 128 BE: 35 patients with 1 BE, 17 with 2 BE and 15 with ≥3 BE; whereas under treatment, 41 developed 62 BE: 28 patients with 1 BE, 8 with 2 BE and 5 with ≥3 BE, including 22 patients with BE before and under ERT/SRT.

**Figure 3:**
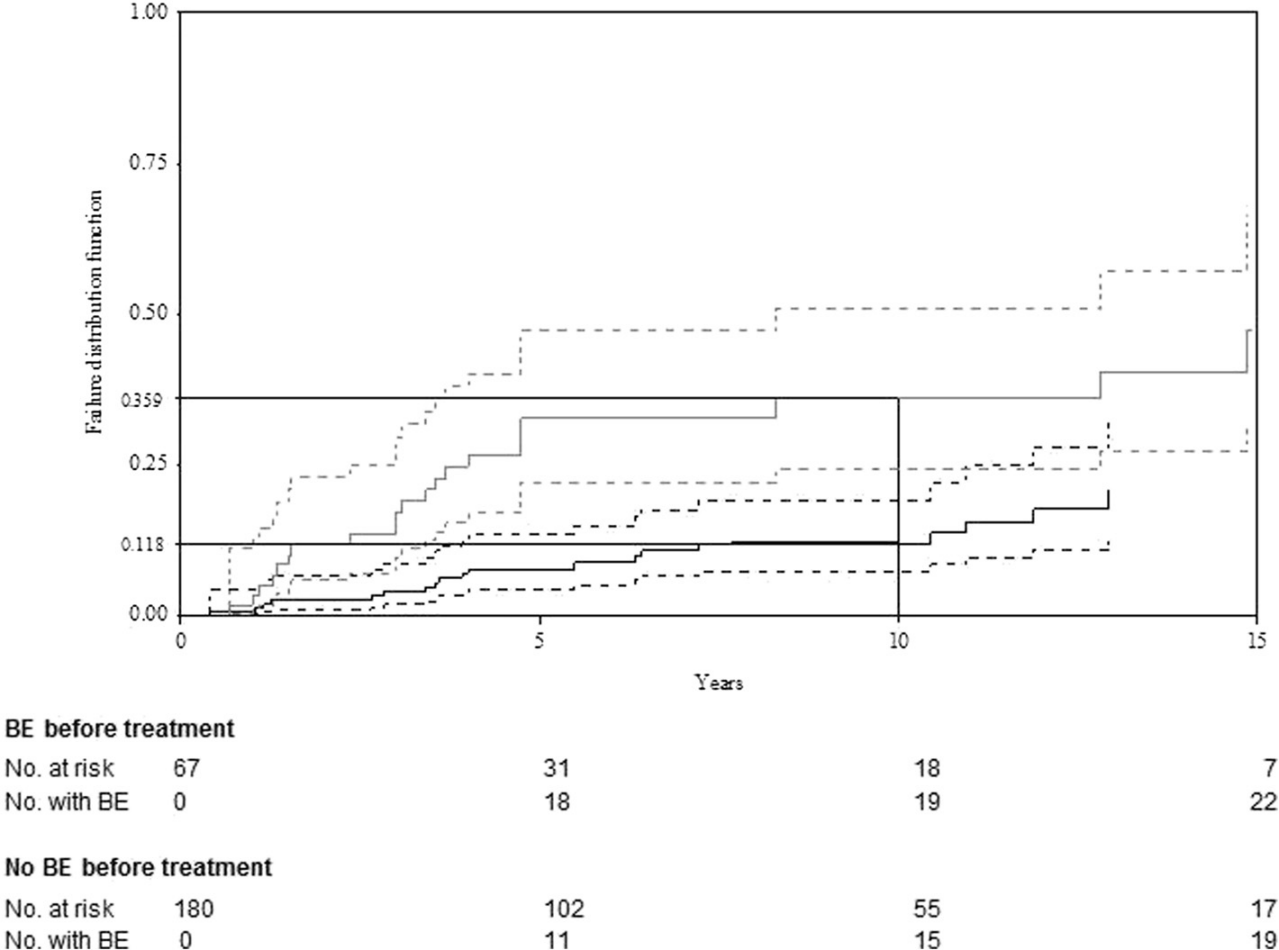
**Impact of BE before treatment on BE occurrence under ERT/SRT for 247 treated GD patients.** The solid **bold grey** line represents patients without BE before treatment; the solid **bold black** line represents the times to first BE. Dashed lines represent the 95% CI of those curves; the estimated probability of BE occurrence after 10 years reported on the y-axis.

The probabilities (95% CI) of BE occurring by 10 years before ERT/SRT were 11.5% (3.1%–19.8%) versus 24.9% (16.6%–33.2%) for age at diagnosis ≤15 years and **>**15 years, respectively (p=0.047). No other covariates were found to influence BE occurrence before ERT/SRT. During treatment, the probabilities (95% CI) of experiencing BE by 10 years were: 11.8% (5.9%–17.6%) and 35.9% (22.2%–49.5%) for patients without or with BE (Figure 3) before ERT/SRT, respectively, with an HR of 9.8 (5.9–16.3) (p<0.001); 29.1% (16.1%–42.1%) versus 14.7% (8.3%–21.1%) with and without splenectomy, respectively, with an HR of 2.1 (1.5–2.9) (p=0.005); 34.9% (29.8%–40.1%) vs 50.3% (46.1%-54.4%) for age at diagnosis ≤15 years and **>**15 years, respectively, with an HR of 0.7 (0.5–0.98) (p=0.04); and 22.3% (15.1%–29.5%) versus 8.1% (0%–17.5%) for diagnosis-to-treatment interval >2 and ≤2 years, respectively, with an HR of 0.5 (0.3–0.9) (p=0.01). Age at diagnosis >15 years was not significantly associated with BE under treatment (p=0.54). In the multivariate analysis with backward elimination, BE before treatment was the only significant risk factor retained.

Table 3 reports ERT/SRT prescribed according to the supply-shortage dates for the 247 recently treated patients. The first treatment prescribed was imiglucerase for 185 patients, alglucerase for 50, miglustat for 7, velaglucerase for 4 patients and taliglucerase for 1. The median diagnosis-to-treatment interval was 9.2 (0.0–47) years for all patients, but only 1.5 (0.0–16) years for patients diagnosed after 1991. The median treatment duration was 7.8 (0–18.3) years. During the supply shortages (June–August 2009), 106 patients discontinued their treatment, 9 switched to miglustat and 46 reduced their imiglucerase doses. Two patients received a combination of miglustat and imiglucerase, which was continued during the shortage (counted only under imiglucerase in Table 3).

**Table 3:** Numbers of recently treated patients given each therapeutic option during ERT shortages according to date

**ERT/SRT**	**2009/06/01**	**2009/06/30**	**2009/08/30**	**2010/04/01**	**2010/12/31**
Imiglucerase*					
>15 years	176	153	71	105	98
≤15 years	32	32	25	27	28
Velaglucerase	–	–	–	–	45
Taliglucerase	–	–	–	–	24
Miglustat	11	14	20	22	21
Without treatment	28	48	131	93	31
Total	247	247	247	247	247

Note that recent refers to 2009–2010, i.e., the last 2 years.

*Only imiglucerase was prescribed to adults (>15 years) or children (≤15 years).

## Discussion

To date, no other publication has analyzed the comprehensive data entered in the FGDR for 562 patients, minus 3 who refused to participate and 97 who died, leaving 465 patients (among 65.8 million inhabitants), yielding prevalence of 1/140,000 inhabitants in France, a number that is probably underestimated. Concerning the entire cohort, although type 1 predominated (85%), types 2 and type 3 represented 4% each, along with 37 (6.9%) perinatal–lethal type. Moreover, the type-2 incidence was the same as that of type 3 but its prevalence was low because of its associated early mortality. The recent publication on the exhaustive Spanish registry [26] reported data similar to ours, with 88.3% type 1, 6.7% type 2 and 5% type 3. Our birth incidence (1/50,000) was higher than previously reported for the GD frequency in non-Jewish populations from EU countries [25,39,40], with a prevalence (1/136,000), close to that of the Spanish registry (1/149,000) [26].

Bone-marrow aspiration (or biopsy) remained the most common laboratory test (57%) providing the GD diagnosis. It is usually the first-line analysis when thrombocytopenia is associated (or not) with splenomegaly and there is no reason to think of immune thrombocytopenia purpura. It is not mandatory and should not be done if the GD diagnosis has been established by enzymatic assay or is already strongly suspected (e.g., possible family history). Rarely, bone-marrow aspiration was considered “normal” but another sample contained the characteristic GD cells.

Fourteen (3.7%) of our 378 followed patients had PD, reaching a prevalence comparable to that reported by Bultron et al. [41]. MG and polyclonal gamma globulinemia occur frequently in GD [42-45]. Among the 378 followed patients, 46 (12.2%) had MG, a rate within the previously reported range (1% [42] to 35% [45]), and median gamma globulinemia at ERT/SRT onset in recently treated patients was 21.7 g/L. Usually, MG is unaffected by ERT [43,44]. However, for patients whose MG was diagnosed under treatment, no pretreatment evaluation was available, and MG had probably been present at treatment onset.

Before 1991, splenectomy was the only available treatment but, since then, it should not have been performed (albeit with exceptions) as a GD treatment. However, it has been used sometimes as a diagnostic tool when splenomegaly and thrombocytopenia coexisted, but should no longer be. Fourteen splenectomies were done after 1991 and after GD diagnosis, usually for patients with splenic complications (splenic infarcts, spleen rupture or large fibrous splenomegaly not amenable to ERT) or a mistaken indication.

BE are the most serious GD complications. They are usually prevented by ERT/SRT, with substantial attenuation of bone pain, bone crises and bone-mineral density [46], although the BE decrease is difficult to evaluate without randomized placebo-controlled trials. In addition, the definition of BE is not homogeneous across studies. Apparently, ERT/SRT does not prevent all BE, as indicated by the estimated respective probabilities of BE occurring by 10 years before and during treatment of 20.3% and 19.8%. It is likely that patients on ERT/SRT would probably have had more complications had they not been treated. Furthermore, we showed that BE before treatment increased the risk of BE under ERT/SRT and was the only factor retained in our multivariate analysis. Note that, as reported by Mistry et al. [38], our univariate analyses also found splenectomy and treatment >2 years after GD diagnosis to increase that risk, while sex and age at diagnosis ≤15 years were associated with increased risk of BE before but not under ERT/SRT. Thus, BE persist as a problem that is not fully resolved by treatment. The continuing challenges remain how to identify patients at risk before and under ERT/SRT, and then to decide whether or not these patients would benefit from earlier treatment onset and/or dose intensification.

In summary, the FGDR strong points are its comprehensiveness, independence, accreditation and/or certification by the various health authorities and cooperation generated among the different French centers. This registry also had to manage the imiglucerase shortage, when more severe GD and children were accorded priority treatment. The FGDR also enabled, during that shortage, nationwide management of the ERT/SRT stock and selection of those patients most in need of therapy (velaglucerase and taliglucerase). In France, GD-patient management is organized so that patients receive treatment near their homes, which improves their quality of life. Even though monitoring is not centralized, the FGDR identification and tracking of patients should contribute to improving their specific care management.

## Abbreviations

ACE: Angiotensin-converting enzyme; BE: Bone events; CI: Confidence interval; EGDTC: Evaluation of Gaucher-Disease Treatment Committee; ERT: Enzyme-replacement therapy; FGDR: French Gaucher Disease Registry; GD: Gaucher’s disease; HR: Hazard ratio; IQR: Interquartile range; MG: Monoclonal gammopathy; PD: Parkinson’s disease; RCLD: Referral Center for Lysosomal Diseases; SRT: Substrate-reduction therapy; TRAP: Tartrate-resistant acid phosphatase.

## Competing interests

Research grants from Genzyme France to University Paris–Diderot to fund statistical analyses and to AP–HP to finance data acquisition. Research grant from Shire France to APRIMI (Beaujon Hospital’s association) to finance data acquisition. C. Serratrice, L. Rossi-Semerano and B. Grosbois, received consulting fees, speaking fees, and/or honoraria from Genzyme (less than $10,000). C. Serratrice, D. Heraoui, F. Camou, A. Masseau and B. Grosbois received consulting fees, speaking fees and/or honoraria from Actelion (less than $10,000). C. Serratrice, F. Camou and B. Grosbois received consulting fees, speaking fees, and/or honoraria from Shire (less than $10,000).

## Authors’ contributions

JS, FM, MV, NB, OF and BF designed research analyzed and interpreted data. All authors, except for FM, MV and DH were involved in treating patients and collecting data. JS had full access to all of the study data and takes responsibility for their integrity and the accuracy of the data analysis. DH developed the original software for the FGDR. JS, MV, NB and FM wrote the draft of the paper, which was then corrected and approved by all authors. All authors read and approved the final manuscript.
